# Diagnoses and visit length in complementary and mainstream medicine

**DOI:** 10.1186/1472-6882-10-3

**Published:** 2010-01-25

**Authors:** Phil JM Heiligers, Judith de Groot, Dick Koster, Sandra van Dulmen

**Affiliations:** 1 NIVEL (Netherlands institute for health services research), P.O. Box 1568, 3500 BN Utrecht, The Netherlands; 2 Utrecht University, Faculty of Social Sciences, Dept. of Work & Organizational Psychology, P.O. Box 80.140, 3508 TC Utrecht, The Netherlands; 3 Comprehensive Cancer Centre North East, P.O. Box 330, 9700 AH Groningen, Groningen, The Netherlands; 4 Homeopathic practice, Theda Mansholtstraat 5a, 2331 JE, Leiden, The Netherlands

## Abstract

**Background:**

The demand for complementary medicine (CM) is growing worldwide and so is the supply. So far, there is not much insight in the activities in Dutch CM practices nor in how these activities differ from mainstream general practice. Comparisons on diagnoses and visit length can offer an impression of how Dutch CM practices operate.

**Methods:**

Three groups of regularly trained physicians specialized in CM participated in this study: 16 homeopathic physicians, 13 physician acupuncturists and 11 naturopathy physicians. Every CM physician was asked to include a maximum of 75 new patients within a period of six months. For each patient an inclusion registration form had to be completed and the activities during a maximum of five repeat visits were subsequently registered. Registrations included patient characteristics, diagnoses and visit length. These data could be compared with similar data from general practitioners (GPs) participating in the second Dutch national study in general practice (DNSGP-2). Differences between CM practices and between CM and mainstream GP data were tested using multilevel regression analysis.

**Results:**

The CM physicians registered activities in a total of 5919 visits in 1839 patients. In all types of CM practices general problems (as coded in the ICPC) were diagnosed more often than in mainstream general practice, especially fatigue, allergic reactions and infections. Psychological problems and problems with the nervous system were also diagnosed more frequently. In addition, each type of CM physician encountered specific health problems: in acupuncture problems with the musculoskeletal system prevailed, in homeopathy skin problems and in naturopathy gastrointestinal problems. Comparisons in visit length revealed that CM physicians spent at least twice as much time with patients compared to mainstream GPs.

**Conclusions:**

CM physicians differed from mainstream GPs in diagnoses, partly related to general and partly to specific diagnoses. Between CM practices differences were found on specific domains of complaints. Visit length was much longer in CM practices compared to mainstream GP visits, and such ample time may be one of the attractive features of CM for patients.

## Background

The supply and demand of complementary medicine (CM) is increasing worldwide [1-7]. By definition CM provides complementary or additional care next to mainstream health service [8]. Not only sick patients but also healthy people with preventive intentions towards their personal health are using CM services. Most empirical work in CM practice focuses on CM use and patients' motives and preferences for seeking CM [4,6,9-12]. Given the growing interest in CM it becomes increasingly important to find out what CM physicians actually do.

Next to the focus on patient utilization the issue about what CM physicians do is found in evaluations of CM treatments by using experimental and observational methods [13]. Further on, an integration debate has started with ideas about the professionalization process for CM [13], propositions for state licensing and certification of CM practitioners are worked out in the USA [14] and European regulations on medicinal products of CM and mainstream medicine are harmonized [15]. However, among CM practitioners all these actions raise concerns about consequences in terms of losing the specific position or being overruled by mainstream medicine [14].

Meanwhile, we also observe developments in mainstream medicine, like stimulating patients' self-management and empowerment [16], which increasingly resemble CM principles such as patients' self-healing power and abilities [17]. Besides, the number of conventionally trained physicians who specialize in CM is growing [17]. These observations might suggest a gradual integration of CM in mainstream medicine [18]. However, in the Royal Dutch Medical Association (RDMA) discussions are currently taken place on health risks as a consequence of the absence of an evidence base of CM interventions. According to the RDMA, it is a physician's responsibility to emphasize the importance of mainstream treatment at all times [19]. In this debate it is emphasized that CM exists outside the world of mainstream medicine. Therefore, it is not only interesting to know what CM physicians do, but also to what extent CM medical activities differ from mainstream activities and also what differences can be traced between different types of CM. Evaluative research on CM practices has lagged behind research on mainstream medicine and in this study we highlight a few points of comparison.

The present paper has several aims. Firstly, a comparison will be made of diagnoses in CM practice and general practice. Since CM physicians are familiar with the nomenclature of mainstream medicine, both CM and mainstream physicians can diagnose their patients using the same terms. Earlier it was found that patients visit CM practices for specific health problems in addition to the use of mainstream health care [20-23]. It was found that chronic disease and poor physical fitness were independently related to CM use [20,21] and also problems like anxiety and depression [22]. Based on physician's diagnoses the current study might confirm a similar picture in the Netherlands. But, if no large differences exist in diagnoses between CM physicians and GPs, we suppose that patients might attend CM physicians primarily for a second opinion or for getting advice from a different angle. Moreover, we may find differences in diagnoses between different types of CM practices, which would imply that each type of CM practice has its own area of interest. Secondly, the length of medical visits in CM and general practice will be compared. Time is known to be a basic commodity in health care. Patients are often left with the feeling that a physician has dedicated not enough time to them [24]. Physicians report the dilemma whether to offer high quality care or to be more efficient and see more patients [25]. Increased visit length is therefore strongly related to higher patient satisfaction [26,27]. Previous research shows that CM physicians spend significantly more time with each patient because they investigate a larger variety of health issues [28].

In summary, the present paper addresses the following two research questions:

1. What complaints are diagnosed in CM practices and do these differ from mainstream general practice?

2. What is the difference in visit length between CM practices and general practice?

More specifically, this paper examines the activities of regularly trained physicians specialized in three different CM specialties in the Netherlands: homeopathy, acupuncture and naturopathy. Homeopathy is based on the idea that in illness the balance in the energy of our organism is disturbed. A substance which has basically a similar pattern of disturbed energy symptoms in its toxic picture as the disturbing illness of the patient will have a healing and stimulating effect. The used substance has to be diluted with alcohol and water in order to be less toxic and still effective [8,29,30]. Acupuncture is based on Traditional Chinese Medicine and is focused on restoring energy flows by using needles which are placed on specific points in patients' bodies to stimulate and restore energy flows. After restoration of energy flows the natural resistance towards illness increases and self-regulating power of the body improves [8,31]. Naturopathy is also based on the idea of self-healing capacities of organisms. The natural balance is disturbed if illnesses emerge. Naturopathy is aiming to restore the natural balance by detoxification. It uses therapeutic methods like neural therapy, phytotherapy and lifestyle and diet-advices, next to natural resources [8,32]. Some CM physicians are also participating in regular practice, most often as GPs. Partly, the initiative for this study started among homeopathic physicians, organised in a registration-network within the Netherlands association for homeopathy physicians (VHAN). From their registration tasks, used for intervision and permanent education, several questions derived which were included in this study.

Patients can visit their CM physician directly or after referral from their GP, but health insurance companies differ in covering the fees for CM. Some costs are not covered or are not mentioned in the policy conditions. Only patients with an additional assurance receive a partial or even 100% reimbursement.

In the Netherlands more than 300 regularly trained physicians work in homeopathy practices, registered by the Netherlands Association for Homeopathy Physicians [33]. All registered homeopathy physicians finished a part-time training in homeopathy during several weekends spread over three years. Every five years all homeopathy physicians have to re-register, which means they have to keep their knowledge up-to-date by attending several training subjects.

In acupuncture practices 200 regularly trained physicians are working and registered by the Netherlands Association for Acupuncture Physicians [34]. The acupuncture education also takes three years, with around twenty days of training and exercise per year.

In naturopathy there are 75 naturopathic physicians, organised within the Netherlands Association of Physicians in Naturopathy [35]. They have all attended a two years program with about 5 weekends of training every year, with several days on specific topics in the end.

All CM training programs are completed with exams and practice exercises. Further on, regularly there are symposia and conferences where new or additional knowledge is shared with the members of the national associations mentioned above.

## Methods

### Selection of CM physicians

The three CM specialties to which the invited physicians belong have the largest numbers of practitioners in the Netherlands. The national organizations of homeopathic physicians (VHAN), physician acupuncturists (NAAV) and naturopathic physicians (ABNG-2000) have assisted in contacting potential participants. For this purpose, all members of the national organizations, i.e. 317 homeopathic physicians, 191 physician acupuncturists and 77 naturopathy physicians, were invited to participate. We opted for 20 practices in each CM specialty and in each practice an inclusion of 75 new patients in a period of six months. New patients are those who attend their CM physician for an initial visit.

Initially, 51 physicians volunteered to participate, but only 44 physicians consented after they had been instructed about the whole procedure. This means that of all registered Dutch CM physicians participating in this study, 5% of all homeopathic physicians, 7% of all physician acupuncturists and 14% of all naturopathic physicians were included. Together, these CM physicians registered 1839 new patients for this study: 502 in homeopathy, 808 in acupuncture and 529 in naturopathy.

### Procedure for registrations and inclusion of patients

All participating physicians were visited by a member of the research team for instructions about the registration forms and procedure. They were asked to include every new patient in the following 6 months up to 75 patients. For each new patient an inclusion registration form had to be completed and during a maximum of 6 months all repeat visits of the included patients were registered up to a maximum of five visits per patient (Table 1), yielding a total of 5919 CM visit registrations.

**Table 1:** Registrations of inclusion and repeat visits (data in this study)

CAM physicians	Inclusion visits (new patients in 6 months)	Repeat visits (max. 5 per patient)	All registered visits during 6 months (max. 6 per patient)
Homeopathy physicians (N = 16)	502	890	1392
Acupuncture physicians (N = 13)	808	2373	3181
Naturopathy physicians (N = 11)	529	817	1346

Totaal (N = 40)	1839	4080	5919

Before the initial visit new patients received a letter with information about the study and they were asked whether they agreed on being included for registrations anonymously. Every patient attending for an initial visit, so not for repeat visits, was included as a new patient.

In the inclusion registration, data were gathered about socio-demographic characteristics of the new patient, the length of the first visit and diagnoses. CM physicians were asked to give their diagnoses using CM-specific concepts as well as following the mainstream ICPC classification [36].

All but the socio-demographic characteristics were again registered after every repeat visit. The study was carried out according to Dutch privacy legislation. The privacy regulation was approved by the Dutch Data Protection Authority. According to Dutch legislation, approval by a medical ethics committee was not required for this observational study.

### Statistical analysis

In this study data gathering is related to two levels in the population: physicians and patients. Multilevel analysis had to be used, because the data are hierarchically structured. Total variation in dependent variables is divided into one part due to differences between patients, and one part due to differences between physicians [37,38]. By using multilevel analysis we could control for patient characteristics: age, gender and education, because differences in these characteristics between patients of CM and general practice (GP) were expected [39]. For the analyses the MLwiN software package was used [40].

### Mainstream data in general practice

The CM registration forms are modified versions of the registration forms used in the second Dutch national survey in general practice (DNSGP-2, 2001) which provided the mainstream general practice data with which the CM practices in the current study were compared [41]. GPs who participated in the DNSGP-2 were representative for the population of Dutch GPs. In the DNSGP-2 data of 151 GPs were included (2% of all GPs). These GPs registered diagnoses and visit length in 2628 patient visits. The mainstream data were collected in 2001 by NIVEL, the institute where two authors of the current study are employed. Some parts of the DNSGP-2, which were used for comparisons in the current study, were developed by one of the authors. The original mainstream data could be used and compared to the CM data collected specifically for this study.

## Results

### Characteristics of physicians and patients

Sixty percent of the participating CM physicians were male, mainly related to the majority of men among homeopathic and naturopathic physicians, whereas women were overrepresented among physician acupuncturists. Most CM physicians were between 40 and 64 years of age with an average of 53 years for male and 50 years for female physicians. On average, male GPs belonged to the same age range, although female GPs were younger: most of them were between 35 and 45 years of age.

The CM patients included in this study differed from those in mainstream GP (Table 2); CM patients were more often female, had a higher educational level and patients visiting a homeopathic physician appeared to be younger.

**Table 2:** Patients in intake registrations of CM physicians compared to registration in GP practices

Patient characteristics	Homeopathy N = 502	Acupuncture N = 808	Naturopathy N = 529	General practice^1^ N = 2628	p-value
Gender (% women)^2^	72.5	69.4	73.4	58.9	^2^<.001
Age*	39.5 ^3,4,5^	46.5	46.0	45.1	^3^<.001 ^4^<.01 ^5^<.001
Educational level (range 1-4)^2^	3.0	3.2	3.3	2.7	^2^<.001
Ethnicity (% immigrants)	2.2 ^3,4,5^	6.9	8.9	5.4	^3^<.05 ^4^<.01 ^5^<.05

*date of calculation January 1, 2008

^1 ^data from the video observation part of the DNSGP-2 in 2001

^2 ^significant difference between CM-patients and patients in general practice

^3 ^significant difference between patients of a specific CM-practice and patients in general practice

^4 ^significant difference between homeopathy and naturopathy patients

^5 ^significant difference between homeopathy and acupuncture patients

### Diagnoses in CM and GP practices: a comparison

The primary diagnoses indicate that CM patients visited CM practitioners for general complaints (as coded in the ICPC) more often than patients in general practice (Table 3), especially for fatigue. More specifically, in homeopathy practices 77% of these general complaints concerned fatigue, in acupuncture practices this percentage was 68%, and in naturopathy practices 45%. In homeopathy and acupuncture practices allergic reactions came as second most frequently diagnosed general complaint, in 12% and 11% of the general complaints, respectively. In naturopathy practices, infections were the second most frequently diagnosed general complaints (12%). Also, psychological problems were diagnosed more often in CM practices than in GP practices, in acupuncture and homeopathy practices about three times more often than in GP practices. The incidence of problems with the nervous system was also found to be higher in CM practices than in GP practices, whereas problems in the cardiovascular system were more often diagnosed in GP practices. Differences in diagnoses between three types of CM practices gave an idea of the specific expertise of each CM specialty. We found that the diagnoses of problems with the musculoskeletal system were highest in acupuncture practices and those of skin problems diagnoses were highest in homeopathic practices. Naturopathic physicians diagnosed more often gastrointestinal problems, compared to GPs as well as the other two CM specialties

**Table 3:** Primary or main diagnoses^1^, first inclusion diagnoses for new patients in CM practices, compared to primary diagnoses in practices of general practitioners in the Netherlands (Multilevel regression analyses)*

	Hom. % (N = 502)	Acup. % (N = 808)	Natur. % (N = 529)	GPs % (N = 2628)**	Intraclass correlation***	p-value
general complaints^2^	15.5	10.1	12.1	4.5	10.1%	^2^<.0005 (all)
blood and blood vessels	0.0	0.0	2.1	1.2	(no test, too few cases)	
gastrointestinal system	6.0 ^4^	6.9 ^5^	16.2^3^	5.3	4.6%	^3,4,5 ^<.0005 (all)
eye	0.0	0.6	0.7	3.5	(no test, too few cases)	
ear	1.1	1.1	1.0	2.4	11.8%	
cardiovascular system ^2^	1.0	1.0	2.0	6.0	13.7%	^2 ^<.0005 (all)
musculoskeletol system	6.9 ^3,6^	22.3 ^5^	6.5^3^	17.4	7.3%	^3,5,6 ^<.0005 (all)
nervous system ^2^	5.9	8.2	4.4	2.0	14.6%	^2gp-h ^.001;^2gp-a ^<.0005;^2gp-n ^.05
psyche	12.3^3^	14.1^3,5^	6.3	3.9	12.3%	^3 ^<.0005 (all);^5 ^.02
respiratory organs	7.5	6.4	5.5	8.9	5.4%	
skin and subcutaneous tissue	14.6 ^3,6^	5.5^5^	11.4	7.9	6.8%	^3 ^.003;^5 ^.01; ^6 ^<.0005
endocrine glands, metabolism, food	1.3	1.3	2.6	1.0	34.9%	
urinary tract	0.7	0.08	0.1	0.04	(no test, too few cases)	
pregnancy, childbirth, contraception	0.0	0.1	0.02	0.1	(no test, too few cases)	
female genital	3.0^3^	1.1	2.2	1.0	19.9%	^3 ^.005
male genital	0.4	0.5	1.3	0.7	(no test, too few cases)	
social problems	0.6	0.2	.04	.02	(no test, too few cases)	

*Controlled for age, gender and education level of patients

** Data from the DNSGP-2 patient registration in 2001(sub dataset for observation study)

*** % of total variance due to differences between doctors

^1^Incidence-data: 6 month in CM practices (2007), 12 month in General Practice (2001)

^2 ^significant difference between all CM-diagnoses and diagnoses in general practice

^3 ^significant difference between a single CM-diagnoses and diagnoses in general practice

^4 ^significant difference between homeopathic - diagnoses and naturopathy diagnoses

^5 ^significant difference between acupuncture- diagnoses and naturopathy diagnoses

^6 ^significant difference between homeopathic - diagnoses and acupuncture diagnoses

### Visit length in CM and GP practices

After the intake visit, physicians registered repeat visits of each patient to a maximum of five within a period of up to 6 months. Of course, not all patients were invited for repeat visits. In acupuncturist practices 320 patients had a maximum of five visits, which corresponds to 39.6% of all registered initial visits. In homeopathy and naturopathy practices, only 35 and 33 patients were invited for a fifth visit, which is 7% and 6.2% of all initial visits, respectively. We found differences between CM practices and general practice concerning visit length (Table 4). In general practice, the visit length varied between 1 and 15 minutes. In CM practices we found initial visit length of 46-60 minutes and even over 60 minutes. Only in acupuncturist practice initial visits were shorter on average, predominantly in the category of 31-45 minutes. Repeat visits lasted between 16 and 30 minutes and even over 30 minutes in CM practices. Repeat visits in acupuncture were longer than in homeopathy, respectively almost 30 minutes against slightly more than 15 minutes. The visit length in general practice did not differ between initial and repeat visits. In CM practice, visit length of initial and repeat visits appeared to be longer with older, higher educated and female patients.

**Table 4:** Registered visit length: a comparison between inclusion and repeat visits of CM physicians and in general practice (Multilevel regression analyses)*

Visit length	Homeopathy (N = 502)		Acupuncture (N = 808)		Naturopathy (N = 529)		General practice (N = 2628)**		p-value
	Mean	SD	Mean	SD	Mean	SD	Mean	SD	
Inclusion visit^1,2^	4.54^6^	0.07	3.52^5^	0.08	4.33	0.08	1.11	0.03	^2 ^<.0005;^5 ^<.0005; ^6 ^<.0005
First repeat visit^1,2^	2.35^6^	0.08	2.86	0.09	2.73	0.1	1.11	0.03	^2 ^<.0005; ^6 ^<.0005
Third repeat visit^1,2^	2.34^6^	0.09	2.87	0.09	2.75	0.1	1.11	0.03	^2 ^<.0005; ^6 ^<.0005
Fifth repeat visit^1,2^	2.55	0.1	2.89	0.09	2.58	0.1	1.11	0.03	^2 ^<.0005

*Controlled for age, gender and education level of patients

** Data from the DNSGP-2 patient registration in 2001(sub dataset for observation study)

^1 ^Measure of visit length: 1 = 1-15 minutes; 2 = 16-30 minutes; 3 = 31-45 minutes; 4 = 46-60 minutes; 5 = > 60 minutes

^2 ^significant difference between all CM visit length and visit length in general practice

^3 ^significant difference between a single CM visit length and visit length in general practice

^4 ^significant difference between homeopathic visit length and naturopathy visit length

^5 ^significant difference between acupuncture visit length and naturopathy visit length

^6 ^significant difference between homeopathic visit length and acupuncture visit length

## Discussion

The aim of this study was to increase our insight in CM physicians' basic medical activities and to compare these activities with those of mainstream general practitioners. For this purpose we used the physician registrations of first inclusion and repeat visits gathered in a period of six months by forty conventionally trained physicians specialized in either homeopathy, acupuncture or naturopathy. Results of these registrations were compared with those from comparable registrations in mainstream general practice (DNSGP-2).

In CM, general complaints - as coded in ICPC - appeared to be more often diagnosed, especially fatigue, allergic reactions and infections, next to psychological problems and problems with the nervous system. The relatively high prevalence of fatigue may be related to the earlier reported patients' need for seeking CM on specific problems or for a second opinion [20-22,42,43], because fatigue is a complex condition [44], which may profit from a holistic approach. In addition, several CM specific diagnoses were registered. The participating physician acupuncturists diagnosed more problems in the musculoskeletal system, which was also found by others [43]. Physicians in homeopathy diagnosed more skin problems, which confirms earlier findings [45,46]. And in naturopathy more problems were attributed to the gastrointestinal system. In earlier studies it was found that CM practitioners are visited for specific types of problems, e.g. chronic disease, anxiety, depression and poor physical fitness [20-23], which was confirmed in this study by CM physician's diagnoses. Moreover, differences are not only found between CM and mainstream practice, but also between types of CM practice which implies specific expertise on different domains.

Comparisons of visit length in CM practices and mainstream GP revealed major differences. General practitioners usually invested between 1-15 minutes, whereas CM physicians used at least 30 minutes for repeat visits and even twice as much for intakes. One of patients' reasons for consulting a CM physician is in line with these findings, i.e. the wish to get ample time to talk with the physician [42]. Other studies on visit length have indicated that in mainstream GPs shorter visits were related to discussions about only one or two health issues, whereas in CM more issues were discussed and a higher number of advices were given [28]. Visit length is also found to be positively related to patient satisfaction [24,26,27]. Yet, at the same time, the abundant time investment on the part of the CM physician does raise questions at policy level about health care supply as well as expenditure. Contrarily, in several studies it was found that CM can contribute to cost-effectiveness (Dulmen AM van, Groot de J, Koster D, Heiligers PhJM: Why seek complementary medicine? An observational study in homeopathic, acupunctural, naturopathic and mainstream medical practice, submitted, [47,48]). For instance, if GPs combine mainstream and alternative treatment, they prescribe less medical drugs and refer less to medical specialists in hospitals [48]. Nevertheless, apart from other indicators, the involvement of time should also be incorporated in studies about the cost effectiveness of CM [49,50].

An asset of the present study was that we were able to compare CM data with that of mainstream GP. The study produced a large set of data on how CM physicians operate. We opted for recruitment of 75 patients by 60 CM physicians (20 of each type), but obviously this was not feasible for many CM practices. This was probably due to the fact that many CM practices lacked practice assistance or administrative support. Still, forty participating CM physicians gathered data of 1839 patients. Comparisons on diagnoses between CM and GP services were possible, because both CM and GP physicians used the ICPC codes. However, in-depth comparative analysis on specific interventions was not possible due to the inherent different approaches.

The self-selected sample of CM physicians limits the implications of our results, because comparable characteristics of all Dutch CM physicians were not available. This means that our results should be carefully interpreted as tendencies in the Netherlands. A second point of concern is the time difference in data gathering of DNSGP-2 (2001) and CM (2007). However, DNSGP-2 was the most recent comparable dataset for the purpose of this study and we assess this time difference not to be a serious problem for the type of comparisons we made. Moreover, comparable parts of the DNSGP-2 were conducted and analysed by one of the authors of this current study, since the DNSGP-2 is performed by the institute were two authors are performing their research (NIVEL).

## Conclusions

The findings from the present study show that the diagnosed problems by CM physician differ from those in mainstream GP. In addition, between types of CM practices differences are found in types of diagnoses. A longer visit length as part of the CM approach is one of the dominant motives for patients to visit CM physicians. Clearly, the question what CM physicians actually do and what their activities mean for health care supply and expenditure requires further exploration and comparisons with activities of mainstream care providers, thereby incorporating the extra time investment.

## Competing interests

The authors declare that they have no competing interests.

## Authors' contributions

PH performed the statistical analyses, drafted the manuscript and contributed to all other aspects of the study, JdG visited participating physicians for instructions and contributed in gathering data, TGCK contributed to the acquisition of data and was involved in drafting the manuscript, SvD drafted the design of the study and was involved in the critical revision of the manuscript. All authors have given final approval of the submitted manuscript.

## Pre-publication history

The pre-publication history for this paper can be accessed here:

http://www.biomedcentral.com/1472-6882/10/3/prepub
